# Previous Renal Replacement Therapy Time at Start of Peritoneal Dialysis Independently Impact on Peritoneal Membrane Ultrafiltration Failure

**DOI:** 10.4061/2011/685457

**Published:** 2011-09-29

**Authors:** Luís Oliveira, Anabela Rodrigues

**Affiliations:** ^1^Nephrology Department, CHP-Hospital Santo António, 4000 Porto, Portugal; ^2^Investigation Unit UMIB/UP, Abel Salazar Biomedical Sciences Institute, 4099-003 Porto, Portugal

## Abstract

*Background*. Peritoneal membrane changes are induced by uraemia per se. We hypothesise that previous renal replacement therapy (RRT) time and residual renal function (RRF) at start of peritoneal dialysis impact on ultrafiltration failure (UFF). *Methods*. The time course of PET parameters from 123 incident patients, followed for median 26 (4–105) months, was evaluated by mixed linear model. Glucose 3.86% solutions were not used in their standard therapy. Sex, age, diabetes, previous RRT time, RRF, comorbidity score, PD modality and peritonitis episodes were investigated as possible determinants of UFF-free survival. *Results*. PET parameters remained stable during follow up. CA125 decreased significantly. Inherent UFF was diagnosed in 8 patients, 5 spontaneously recovering. Acquired UFF group presented type I UFF profile with compromised sodium sieving. At baseline they had lower RRF and longer previous time of RRT which remained significantly associated with UFF-free survival by Cox multivariate analysis (HR 0.648 (0.428–0.980), *P* = 0.04) and (HR 1.016 (1.004–1.028), *P* = 0.009, resp.). UFF free survival was 97%, 87% and 83% at 1, 3 and 5 years, respectively. *Conclusions*. Inherent UFF is often unpredictable but transitory. On the other hand baseline lower RRF and previous RRT time independently impact on ultrafiltration failure free survival. In spite of these detrimental factors generally stable long-term peritoneal transport parameters is achievable with a 5-year cumulative UFF free survival of 83%. This study adds a further argument for a PD-first policy.

## 1. Introduction

Peritoneal membrane ultrafiltration failure (UFF) is a relevant long-term complication menacing peritoneal dialysis (PD) [1]. It has been reported to lead to technique failure in a rate of 1.7% [2] to 13.7% [3]. Peritoneal morphological changes seem to be related to dialysis solutions, bioincompatibility, and to infections. Uremic milieu per se may also contribute to peritoneal changes since both submesothelial fibrosis and vascular changes are already present in uremic patients, before dialysis induction. The median thickness of the submesothelial compact collagenous zone was 50 micron for normal subjects, but was 140 micron for uremic predialysis patients, 150 micron for patients undergoing hemodialysis, and 270 micron for patients undergoing PD [4]. Honda et al. concluded that the average peritoneal thickness was increased in uremic patients and progressively thickened as the duration of peritoneal dialysis prolonged, while the lumen/vessel diameter ratio was lower in uremia than normal and progressively decreased as the duration of peritoneal dialysis was prolonged [5]. Thus, the effect of uremia on the baseline and time dependent profiles of peritoneal membrane function deserves further studies. It is a continuous bystander in dialysis patients only more recently introduced in PD animal models [6], but often excluded from UFF analysis [7].

 Currently, the determinants of small solutes, proteins, and water transport across the peritoneal membrane, as well as their evolution during PD therapy, are still a matter of debate. Recently, some mechanisms involved in acquired UFF have been identified but less is known about the role of previous renal replacement therapy time in this issue. Moreover, early UFF is still an unexplained phenomenon. 

A fast transport status is the primary mechanism of UFF and it is sometimes documented as an inherent condition whose clinical impact has been debated [8–11] but early UFF still remains often unexplained [7, 12]. Later during PD, loss of glucose osmotic conductance might add to the process of acquired UFF, with a disproportionally more severe compromise of free water transport [13]. Additionally, it is known that peritoneal fibrosis is induced by PD solutions but uraemia per se is also a fibrogenic factor [14]. Residual renal function and precious renal replacement therapy time at PD start are clinical variables that reflect the cumulative uremia stage. 

We aim to identify relevant clinical determinants of early and acquired UFF, focusing on the independent impact of previous renal replacement therapy time and residual renal function at start of PD. Its eventual independent impact may strengthen PD prescription as a first renal replacement therapy option.

## 2. Patients and Methods

We prospectively studied 123 consecutive peritoneal dialysis incident patients enrolled at Hospital Santo António PD Unit since 1st January 2001. All patients were free of hypertonic 3.86% glucose solutions. Standard prescription included low-GDPs solutions; median glucose concentration exposure was 1.65% (range 1.36%–2.27%) and 40% used icodextrin. Age, diabetes, previous renal replacement therapy time (RRT), baseline residual renal function (RRF) quantified as glomerular filtration rate (GFR mL/min/1.73 m^2^)—based on 24 hrs urine collections with determinations of creatinine and urea, Davies comorbidity score, automated PD, and peritonitis events were investigated as possible determinants of baseline or late UFF. All patients performed baseline and yearly 3.86%-peritoneal equilibration tests (PETs), being followed for median 26 (4–105) months: D/P creatinine, D/D0 glucose, sodium sieving, and peritoneal ultrafiltration (UF) were analyzed, and UF failure was defined as a net UF lower than 400 mL after a 4-hour dwell with 3.86%. PET; CA125 appearance rate was also calculated after 4 hours of PET dwell.

The time course of PET parameters was explored by repeated measurements mixed linear model analysis with SPSS software. 

Clinical and laboratory parameters considered to be possible determinants of UFF were investigated and its impact on UFF-free survival was studied by using Cox multivariate analysis. Investigation was made both in the whole cohort and in the subgroup after excluding patients admitted after renal graft failure.

## 3. Results

The investigated patients had a mean age of 48 ± 15 (20–82) years and female predominance (62%). Twenty-three patients (18.7%) were diabetic, thirty (24.4%) were anuric and the majority of them (59%) were on APD. Fifty-four patients (43.9%) had been on previous renal replacement therapy (RRT) for a median time of 63 months (2–410): 30.9% after hemodialysis (HD) and 13.0% after renal transplant failure (RT). 

### 3.1. Time Course of Peritoneal Membrane Function

**Figure 1:**
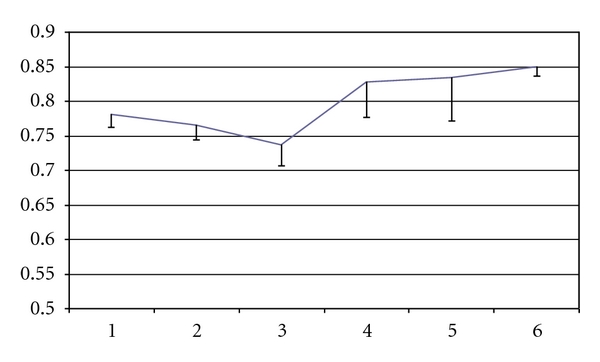
PET 3.86% D/P creatinine means by time (years on PD) estimated by repeated measurements mixed model analysis (*P* = NS).

**Figure 2:**
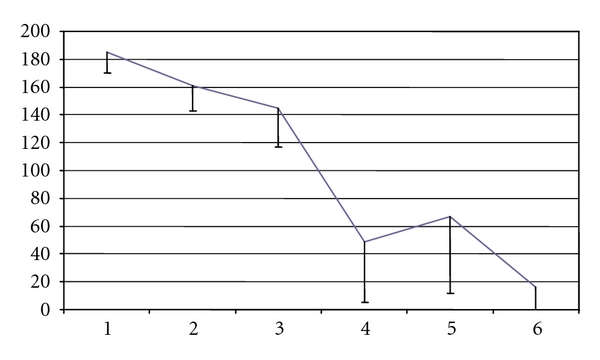
Effluent CA125 U/min means by time (years on PD) estimated by repeated measurements mixed model analysis (*P* = 0.009).

By repeated measurements mixed model analysis, it was shown that small solute, UF, and sodium-sieving parameters remained essentially stable during the followup. A U-shaped curve of D/P creatinine was documented, but this variation with time did not attain significance (Figure 1). CA125 decreased progressively (*P* = 0.009) (Figure 2), mainly in late UFF patients. The same profile was documented in the subgroup of patients after excluding those admitted after renal graft failure (D/P creatinine U-shaped curve though *P* = ns; for Ca125 parameter *P* = 0.015).

### 3.2. Inherent and Acquired Ultrafiltration Failure

UFF was documented in 15 patients: eight patients (6.5%) showed baseline ultrafiltration failure (UFF) while seven patients (5.7%) developed acquired UFF. Notably, five patients completely recovered from baseline UFF.

Sex, age, diabetes, comorbidity score, baseline RRF, and previous RRT did not differ significantly between baseline UFF group and the other patients (Table 1). D/P creatinine of inherent UFF group and other patients was similar (0.74 ± 0.11 versus 0.75 ± 0.13, *P* = 0.74). Also sodium sieving did not differ significantly between the groups (D/P Na60 0.90 ± 0.038 versus 0.87 ± 0.034, *P* = 0.057), although a trend was noticed. 

**Figure 3:**
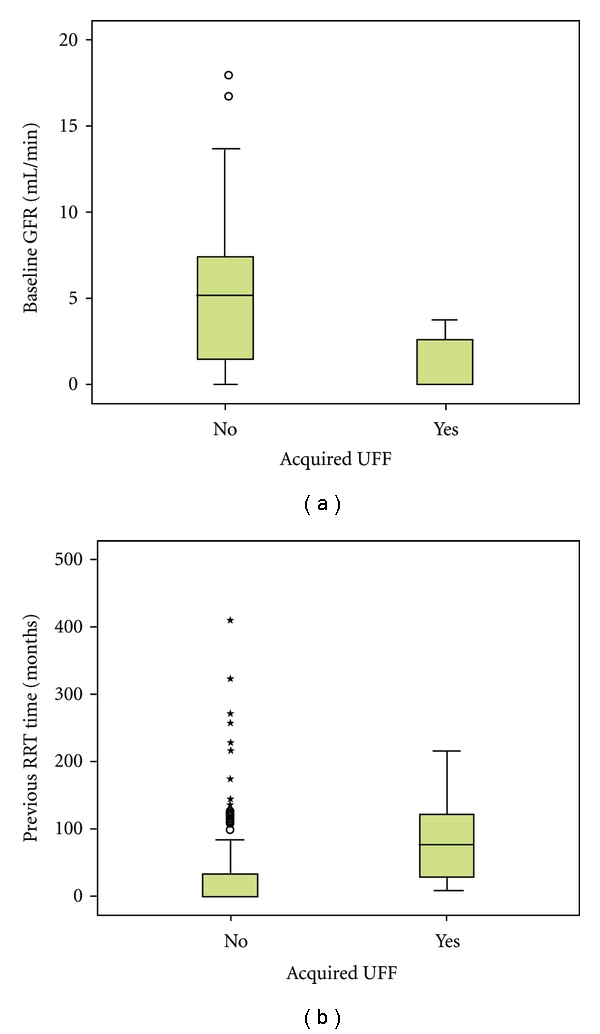
Comparison between acquired UFF patients and preserved UF group: acquired UFF group had significantly lower baseline residual renal function (*P* = 0.009) and longer previous renal replacement therapy (*P* = 0.003).

On the other hand, the acquired UFF group presented type I UFF profile with clearly compromised sodium sieving (D/P creatinine was 0.83 ± 0.10 versus 0.72 ± 0.12, *P* = 0.035 and D/PNa60 0.92 ± 0.028 versus 0.87 ± 0.034, *P* = 0.010) (Table 2). They had significantly lower baseline RRF (*P* = 0.009) and longer previous RRT time (*P* = 0.003) (Figure 3).

### 3.3. Ultrafiltration Failure Free Survival 

#### 3.3.1. UFF-Free Survival Was 97%, 87%, 83% at 1, 3, 5 Years (Figure 4)

Baseline lower RRF and longer previous RRT were independently associated with lower UFF-free survival by Cox multivariate analysis (Table 3). Sex, age, diabetes, APD modality, and peritonitis did not significantly impact on UFF-free survival. After excluding patients admitted after graft failure (*n* = 13), RRT time remained independently associated with UFF (B 0.023 Exp(B) 1.023 (1.007–1.040) *P* = 0.006) as also baseline GFR (mL/min) (B-0.447 Exp(B) 0.64 (0.412–0.993) *P* = 0.047).

## 4. Discussion

Our study highlights that residual renal function and previous cumulative renal replacement therapy time, in a contemporary PD population-free of hypertonic 3.86% glucose solutions exposition, independently impact on ultrafiltration-failure-free survival. This study therefore adds a new argument for a PD-first policy as a strategy to improve technique survival. 

Additionally it documented that important membrane functional changes occur already from start of PD. Measuring peritoneal transport characteristics is an approach which gives objective and reproducible information on peritoneal performance and possible etiological factors of UFF [15]. A fast transport status however, either alone or in combination with other alterations in membrane function, remains the most common underlying mechanism of UFF. We indeed showed that acquired UFF group presented type I UFF profile with compromised sodium sieving. UFF in long-term PD is most often due to a combination of a rapid disappearance of the osmotic gradient, together with an impairment of transcellular water transport (TCWT) [13]. But the activity of water channels is dependent and limited by the crystalloid osmotic pressure [16] which our methodology did not allow to be calculated, being a limitation for characterization of the late stage UFF. In spite of that we were able to document free water transport compromise by the indirect sign of decreased sodium sieving. For this reason, we are now measuring the actual UF and effluent sodium after 60 min dwell followed by effluent reinfusion and completion of standardized 4-hour 3.86% PET which allows evaluation of both free water and standardized small solute transport [17]. Finally, back filtration of fluid through the capillaries and fluid reabsorption from the peritoneal cavity into tissues and lymphatics is a recognized mechanism of UF failure and accounts for approximately 25% of the cases of UF dysfunction, but only investigational methods with tracer macromolecules hard to apply in a clinical ward are able to evaluate this.

More relevant to our study was to highlight that baseline UFF is prevalent but often transitory and not predicted by baseline clinical variables according to previous investigations [7–12]. Many aspects of early stage transport changes and mechanisms indeed remain to be understood. While lymphatic absorption cannot be excluded as a cause of early UFF, the evolution of patients recovering ultrafiltration capacity does not support such etiology. We can speculate that although no significant changes were documented in small solute transport at baseline between the groups with and without UFF, membrane structural changes induced by uremia per se namely interstitium fibrosis might justify the marginal compromise of sodium sieving. This indeed gives lumped information and is not only dependent on an increase of diffusive mass transport coefficients for small solutes, but also on a decrease of the glucose osmotic conductance (number and function of aquaporins, number and diameter of small pores) and on reduction of ultrafiltration coefficient of the peritoneal membrane (role for the interstitium changes).

Interestingly, we found a U-shaped curve of D/P creatinine in the followup, already previously reported by our group and others [8, 13, 18] though not attaining statistical significance in this contemporary cohort. The early phase of D/P creatinine normalisation may express an adaptive process whose mechanisms are unclear but may include early recruitment or vasodilation of vessels mediated by vasoactive mediators, many of them secreted by mesothelial cells. Therefore, in some of our patients a transitory fast transport status may explain the inherent UFF. In other patients, the causes of such baseline UFF are not clear, pointing to the complexity of peritoneal membrane time-dependent functional changes. The risk phase with clinical impact may be documented by the late increasing side of the U-shaped curve, with decreasing mesothelial cell mass as a marker of structural changes that go along with UFF and sodium sieving compromise. Again we highlight the importance of routine membrane monitorization also including an accessible and affordable structural marker—CA125 effluent appearance rate [19]. 

However, our global population presented stability in the transport rates for small molecules and sodium sieving over time. This is in accordance with previous publications where small-solute transport parameters were found to be increased only in long-term patients [20], but happily, in disagreement with the gloomier reports of sustained and inexorable increase of D/P creatinine over time, already from the start [21]. On the other hand, uremia and baseline GFR as its surrogate, is indeed an important bystander not usually taken into account in peritoneal membrane changes investigation. We identified it here as a clinical variable that independently impacts on UFF-free survival. This clue deserves further investigation but suggests that uremia may be crucial to explain acquired peritoneal membrane changes, and although it has not been associated with baseline transport characteristics may modulate membrane time-dependent profile [4–6]. 

As a limitation of our study, we did not control for a panel of pharmacological agents shown experimentally to modulate membrane structure, namely, renin angiotensine system inhibitors and erythropoiesis stimulating agents [22, 23]. However, since the use of these agents is massive in our PD patients, it is not presumed to change our results. 

In spite of some controversy [18], our study also showed that the influence of peritonitis on the development of UFF seems to be limited. It has been found that patients with a history of peritonitis were not different from patients without a previous peritonitis episode in terms of D/P ratio and mass transfer area coefficient of low molecular weight solutes, lymphatic absorption rate, transcapillary ultrafiltration, and net ultrafiltration [24]. Only clusters of peritonitis or peritonitis episodes that occur later in PD have been described as causing a decrease in UF [25]. 

Considering the link between comorbidity and peritoneal transport, data is controversial. Some papers document that systemic inflammation associated with comorbid diseases and elevated interleukin- (IL-) 6 level may induce vasodilation and neoangiogenesis in peritoneal membrane [26]. We did not find any association between morbidity and higher transport rates, like others [27], nor comorbidity score was predictive of UFF.

As a structural marker, effluent cancer antigen 125 can be used reflecting mesothelial cell mass and cell turnover in stable, noninfectious PD patients. Its decrease with the duration of PD, described previously [28], is consistent with the reported cell loss observed in peritoneal biopsies. Such profile of effluent CA125 appearance rate is therefore more likely a sign of damage to the peritoneum than a causative factor of UF by itself. It can be interpreted as an additional prognostic sign, adding to the changes of D/P creatinine and effluent IL-6 [29]. 

In conclusion, this paper documents early-stage peritoneal membrane changes with transitory cases of inherent ultrafiltration capacity failure dissociated from small-solute transport, whose mechanisms remain unclear. On the other hand, lower baseline RRF and previous longer RRT were associated with acquired UFF in our population. In spite of these detrimental factors, we found generally stable long-term peritoneal transport parameters with 5 years 83% cumulative UFF-free survival. By highlighting the importance of previous cumulative RRT time and baseline RRF concerning peritoneal membrane function status these results support a PD-first strategy in the integrated renal replacement treatment plan.

## Figures and Tables

**Figure 4:**
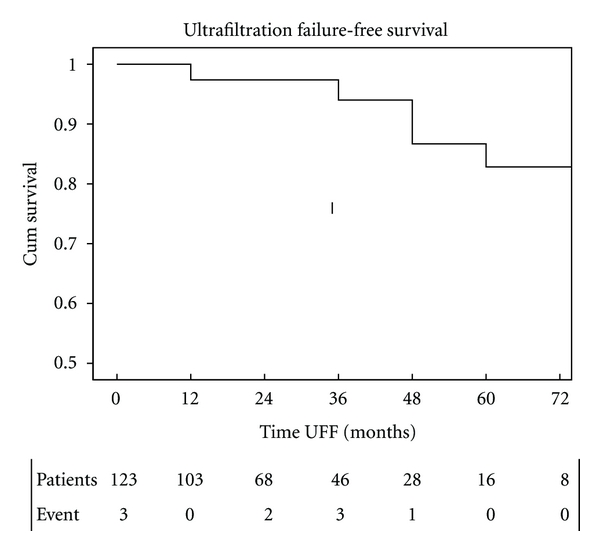
UFF-free survival rate was 97%, 87%, 83% at 1, 3, 5 years.

**Table 1:** Comparison between inherent (baseline) UFF group and baseline-stable patients (categorical data as number (percentage) compared by Fisher's exact test; continuous data presented as median (25%–75% interquartile range), compared by Mann Whitney *U*-test).

	Baseline UFF	Baseline stable group	*P*
	*N* = 8	*N* = 115	
Sex (male) (*N*; %)	3 (37.5%)	44 (38.3%)	1
Diabetes (*N*; %)	1 (12.5%)	22 (19.1%)	1
Age	44 (38–66)	47 (35–60)	0.64
Comorbidity score (≥2)	1 (12.5%)	30 (26%)	0.76
Baseline GFR mL/min	4.2 (2.2–6.2)	4.8 (0–7.4)	0.90
RRT time (months)	0 (0–1.9)	1.7 (0–41)	0.32
PET parameters			
D/P creatinine	0.76 (0.65–0.81)	0.77 (0.66–0.86)	0.74
D/D0 glucose	0.25 (0.21–0.28)	0.29 (0.25–0.3)	0.12
PET drainage	2200 (2100–2287)	2800 (2600–2900)	<0.0001
D/P Na 60	0.90 (0.86–0.93)	0.87 (0.85–0.89)	0.057
Dip Na	0.028 (0.001–0.076)	0.050 (0.022–0.073)	0.21
Ca125 U/min	143 (67–350)	136 (85–241)	0.89

**Table 2:** Comparison between acquired UFF group and stable patients (categorical data as number (percentage) compared by Fisher's exact test; continuous data presented as median (25%–75% interquartile range), compared by Mann Whitney *U*-test); Δ Ca125 is the variation between last evaluation in the followup and baseline effluent CA125 U/min levels.

	Acquired UFF	Stable group	*P*
	*N* = 7	*N* = 108	
Sex (male) (*N*; %)	5 (71.4%)	39 (36.1%)	0.104
Diabetes (*N*; %)	0 (0%)	22 (20.4%)	0.343
Age	39 (34–45)	48 (35–60)	0.179
Comorbidity score (≥2)	2 (28.5%)	28 (25.9%)	0.203
RRT time (months)	77 (13–147)	0 (0–33)	0.003
Baseline GFR mL/mn	0 (0–3.6)	5.1 (1.37–7.4)	0.009
APD (Yes)	5 (71.4%)	61 (56.5%)	0.697
Peritonitis (Yes)	7 (100%)	61 (56.5%)	0.040
Peritonitis (*n*)	3 (1–4)	1 (0–2)	0.008
PET parameters			
D/P creatinine	0.78 (0.75–0.94)	0.71 (0.64–0.81)	0.037
D/D0 glucose	0.24 (0.18–0.30)	0.30 (0.30–0.34)	0.021
PET drainage	2300 (2250–2400)	2800 (2600–2900)	<0.0001
D/P Na 60	0.92 (0.88–0.95)	0,88 (0.85–0.89)	0.007
Dip Na	0.028 (−0.007–0.054]	0.048 (0.021–0.071)	0.16
Ca125 U/min	23 (10–28)	163 (86–227)	<0.0001
Δ Ca125	−52 (−79–−13)	0 (−29–53)	0.004

**Table 3:** Multivariate Cox proportional hazard analysis of variables significantly associated with UFF-free survival. Sex, age, diabetes, APD, and peritonitis did not significantly impact on UFF-free survival.

	B	Exp(B) (95% CI)	*P*
Baseline GFR (mL/min)	−0.434	0.648 (0.428–0.980)	0.040
RRT time (month)	0.016	1.016 (1.004–1.028)	0.009

Cox regression; status acquired UFF.

## References

[1] Bargman JM, Krediet RT, Lo WK (2008). What are the problems with using the peritoneal membrane for long-term dialysis?. *Seminars in Dialysis*.

[2] Maiorca R, Cancarini GC, Zubani R (1996). CAPD viability: a long-term comparison with hemodialysis. *Peritoneal Dialysis International*.

[3] Kawaguchi Y (1999). National comparisons: optimal peritoneal dialysis outcomes among Japanese patients. *Peritoneal Dialysis International*.

[4] Williams JD, Craig KJ, Topley N (2002). Morphologic changes in the peritoneal membrane of patients with renal disease. *Journal of the American Society of Nephrology*.

[5] Honda K, Hamada C, Nakayama M (2008). Impact of uremia, diabetes, and peritoneal dialysis itself on the pathogenesis of peritoneal sclerosis: a quantitative study of peritoneal membrane morphology. *Clinical Journal of the American Society of Nephrology*.

[6] Vrtovsnik F, Coester AM, Lopes-Barreto D (2010). Induction of chronic kidney failure in a long-term peritoneal exposure model in the rat: effects on functional and structural peritoneal alterations. *Peritoneal Dialysis International*.

[7] Selgas R, Bajo MA, Cirugeda A (2005). Ultrafiltration and small solute transport at initiation of PD: questioning the paradigm of peritoneal function. *Peritoneal Dialysis International*.

[8] Rodrigues AS, Martins M, Korevaar JC (2007). Evaluation of peritoneal transport and membrane status in peritoneal dialysis: focus on incident fast transporters. *American Journal of Nephrology*.

[9] Rodrigues AS, Almeida M, Fonseca I (2006). Peritoneal fast transport in incident peritoneal dialysis patients is not consistently associated with systemic inflammation. *Nephrology Dialysis Transplantation*.

[10] Chung SH, Heimbürger O, Lindholm B (2008). Poor outcomes for fast transporters on PD: the rise and fall of a clinical concern. *Seminars in Dialysis*.

[11] Reyes MJF, Bajo MA, Hevía C (2007). Inherent high peritoneal transport and ultrafiltration deficiency: their mid-term clinical relevance. *Nephrology Dialysis Transplantation*.

[12] Selgas R, Bajo MA, Castro MJ (2000). Risk factors responsible for ultrafiltration failure in early stages of peritoneal dialysis. *Peritoneal Dialysis International*.

[13] Parikova A, Smit W, Struijk DG, Zweers MM, Krediet RT (2005). The contribution of free water transport and small pore transport to the total fluid removal in peritoneal dialysis. *Kidney International*.

[14] De Vriese AS, Tilton RG, Mortier S, Lameire NH (2006). Myofibroblast transdifferentiation of mesothelial cells is mediated by RAGE and contributes to peritoneal fibrosis in uraemia. *Nephrology Dialysis Transplantation*.

[15] Van Biesen W, Heimburger O, Krediet R (2010). Evaluation of peritoneal membrane characteristics: clinical advice for prescription management by the ERBP working group. *Nephrology Dialysis Transplantation*.

[16] La Milia V, Limardo M, Virga G, Crepaldi M, Locatelli F (2007). Simultaneous measurement of peritoneal glucose and free water osmotic conductances. *Kidney International*.

[17] Cnossen TT, Smit W, Konings CJAM, Kooman JP, Leunissen KM, Krediet RT (2009). Quantification of free water transport during the peritoneal equilibration test. *Peritoneal Dialysis International*.

[18] del Peso G, Fernández-Reyes MJ, Hevia C (2005). Factors influencing peritoneal transport parameters during the first year on peritoneal dialysis: peritonitis is the main factor. *Nephrology Dialysis Transplantation*.

[19] Rodrigues AS, Silva S, Bravo F (2008). Peritoneal membrane evaluation in routine clinical practice. *Blood Purification*.

[20] Struijk DG, Krediet RT, Koomen GCM, Boeschoten EW, Hoek FJ, Arisz L (1994). A prospective study of peritoneal transport in CAPD patients. *Kidney International*.

[21] Davies SJ, Phillips L, Naish PF, Russell GI (2001). Peritoneal glucose exposure and changes in membrane solute transport with time on peritoneal dialysis. *Journal of the American Society of Nephrology*.

[22] Noh H, Ha H, Yu MR, Kim YO, Kim JH, Lee HB (2005). Angiotensin II mediates high glucose-induced TGF-*β*1 and fibronectin upregulation in HPMC through reactive oxygen species. *Peritoneal Dialysis International*.

[23] Mondello S, Mazzon E, Di Paola R (2009). Erythropoietin suppresses peritoneal fibrosis in rat experimental model. *European Journal of Pharmacology*.

[24] Fußhöller A, Zur Nieden S, Grabensee B, Plum J (2002). Peritoneal fluid and solute transport: influence of treatment time, peritoneal dialysis modality, and peritonitis incidence. *Journal of the American Society of Nephrology*.

[25] Davies SJ, Bryan J, Phillips L, Russell GI (1996). Longitudinal changes in peritoneal kinetics: the effects of peritoneal dialysis and peritonitis. *Nephrology Dialysis Transplantation*.

[26] Pecoits-Filho R, Araújo MRT, Lindholm B (2002). Plasma and dialysate IL-6 and VEGF concentrations are associated with high peritoneal solute transport rate. *Nephrology Dialysis Transplantation*.

[27] Rumpsfeld M, McDonald SP, Purdie DM, Collins J, Johnson DW (2004). Predictors of baseline peritoneal transport status in Australian and New Zealand peritoneal dialysis patients. *American Journal of Kidney Diseases*.

[28] Ho-Dac-Pannekeet MM, Hiralall JK, Struijk DG, Krediet RT (1997). Longitudinal follow-up of CA125 in peritoneal effluent. *Kidney International*.

[29] Sampimon DE, Coester AM, Struijk DG, Krediet RT (2007). Time course of peritoneal transport parameters in peritoneal dialysis patients who develop peritoneal sclerosis. *Advances in Peritoneal Dialysis*.

